# Navigating Treatment-Resistant Major Depressive Disorder With Suicidal Ideation: Exploring the Efficacy of Spravato (Esketamine) in an 86-Year-Old Male

**DOI:** 10.7759/cureus.76090

**Published:** 2024-12-20

**Authors:** Aarman P Jivraj, Katherine A Heimer, Christopher J Wukovits, Justin B Itteera, Annupriya Itteera

**Affiliations:** 1 Psychiatry, Hillside TMS (Transcranial Magnetic Stimulation) and Esketamine, New Hyde Park, USA; 2 Ophthalmology, OCLI (Ophthalmic Consultants of Long Island) Vision, Glen Cove, USA; 3 School of Public Health and Health Professions, University at Buffalo, Buffalo, USA; 4 Mental Health Counseling, Hofstra University, Hempstead, USA; 5 Psychiatry, Creedmoor Psychiatric Center, Queens Village, USA; 6 Psychiatry, Northwell Health, New Hyde Park, USA

**Keywords:** esketamine, geriatrics population, major depressive disorder (mdd), psychiatry and mental health, treatment resistant depression (trd)

## Abstract

This case study examines the novel application of Spravato (intranasal esketamine) to address treatment-resistant Major Depressive Disorder (MDD) in an 86-year-old patient. Notably, this represents one of the oldest documented individuals to be administered intranasal esketamine for a diagnosis of Treatment-Resistant Depression (TRD) alongside suicidal ideation. This case study contributes to the growing body of evidence on its use in elderly populations suffering from MDD with suicidal ideation. Given the limited research on intranasal esketamine therapy in geriatric populations, this study highlights unique pharmacodynamic and clinical challenges specific to this demographic, particularly regarding advanced age, comorbidities, and sensitivity to adverse effects. Under the supervision of a double board-certified adult and geriatric psychiatrist, this case study examines how intranasal esketamine therapy can lead to positive treatment outcomes in geriatric populations. This case study also addresses the safety and efficacy of intranasal esketamine therapy, contributing to the broader discourse on its potential as a viable treatment option for TRD, especially for geriatric patients resistant to traditional therapies. While current literature primarily focuses on individuals aged 65 and younger, this case study aims to provide insights into future applications in geriatric populations to improve existing clinical guidelines.

## Introduction

Major Depressive Disorder (MDD) characterizes a persistently low mood and a significant loss of interest or pleasure in almost all activities, lasting for at least two weeks, representing a change from previous functioning. To meet the Diagnostic and Statistical Manual of Mental Disorders, fifth edition (DSM-5) diagnostic criteria for MDD, an individual must experience at least five of the following symptoms during the same two-week period: (1) depressed mood for most of the day, nearly every day, evidenced by subjective reports or observed by others; (2) markedly diminished interest or pleasure in all, or nearly all, activities; (3) significant weight loss, gain, or a change in appetite; (4) insomnia or hypersomnia; (5) observable psychomotor agitation or retardation; (6) fatigue or loss of energy; (7) feelings of worthlessness or excessive guilt; (8) reduced ability to think or concentrate, or indecisiveness; and (9) recurrent thoughts of death, suicidal ideation, or a specific plan or attempt to commit suicide. Additionally, symptoms must cause clinically significant impairment in social, occupational, or other important areas of functioning (Criterion B). The depressive episode cannot be attributed to the physiological effects of a substance or another medical condition (Criterion C), and the symptoms must not be better explained by another psychiatric disorder (Criterion D). Treatment-Resistant Depression (TRD) is diagnosed when an individual with MDD does not adequately respond to at least two different antidepressant treatments, despite proper dosing and duration [1].

A systematic meta-analysis and review approximated the overall prevalence of depression among geriatric individuals to be approximately 19.2%, highlighting the significant impact of this condition on older adults globally [2]. Geriatric populations also face unique challenges, such as increased medical comorbidity, cognitive decline, and polypharmacy, which complicate the diagnosis and treatment of MDD and TRD. These factors make the impact of these conditions incredibly complex and pressing [3]. Conventional treatment modalities for MDD include psychotherapy and pharmacotherapy. Psychotherapy, specifically cognitive behavioral therapy (CBT), has shown effectiveness in treating depressive symptoms in older adults [4]. However, cultural stigmatization regarding mental health, coupled with communicative barriers like cognitive decline and hearing loss, may prevent effective communication and understanding during therapy sessions, thus reducing overall efficacy rates for older individuals [5]. They may perceive depression as a personal failure, rather than recognizing it as a treatable medical condition, reducing the likelihood of pursuing therapy or seeking pharmacological treatment for depressive disorders.

Pharmacological treatment typically involves the use of oral antidepressants, such as serotonin-norepinephrine reuptake inhibitors (SNRIs) and selective serotonin reuptake inhibitors (SSRIs); however, geriatric populations often experience reduced efficacy and heightened adverse effects from these medications [6]. This is likely due to age-related changes in drug metabolism and increased susceptibility to adverse effects. Consequently, there is a growing need for alternative treatment options suited to the physiological and psychiatric profiles of this demographic. Spravato, also known as intranasal esketamine, is FDA-approved for the treatment of TRD in patients who have not responded to at least two different oral antidepressants. It also has shown effectiveness in alleviating depressive symptoms in adults with MDD, including those with suicidal ideation or thoughts [7]. Notably, a study published in the Journal of Clinical Psychiatry reported a 69.5% response rate in older adults treated with intranasal esketamine; however, targeted research in this age group remains scarce [8]. As an enantiomer of ketamine, Spravato (intranasal esketamine) primarily exerts its effects by antagonizing N-methyl-D-aspartate (NMDA) receptors in the brain, a mechanism distinguishing intranasal esketamine from traditional antidepressant therapy, which typically targets the monoaminergic neurotransmitters. Esketamine's noncompetitive, nonselective NMDA receptor blockade transiently increases glutamate release, stimulating downstream intracellular pathways that improve synaptic connectivity. This action is thought to play a role in the quick effects of antidepressants, which are especially beneficial in the immediate treatment of persistent depressive symptoms [9]. Despite its emerging role in psychiatric treatment, significant research gaps remain concerning its use in geriatric populations. Initial phases of clinical trials of intranasal esketamine primarily involved adults aged 18 to 64, with a limited number of participants aged 80 and older, underscoring the absence of data on the efficacy and safety of intranasal esketamine in geriatric populations [3,10].

This case report discusses the treatment of an 86-year-old male with TRD and suicidal ideation, highlighting the clinical complexities posed by advanced age, medical comorbidity, and an extensive treatment history. It examines how intranasal esketamine provided significant symptom relief after decades of limited success with traditional therapies, emphasizing intranasal esketamine's benefits for geriatric patients. The report emphasizes the need for targeted studies to evaluate intranasal esketamine's pharmacodynamics, optimal dosing, and long-term impacts in this population to refine its use in depression management.

## Case presentation

Patient's medical and psychiatric history

The patient is an 86-year-old married Caucasian male, a retired physician, living with his spouse in a private home. In December of 2023, the patient presented to the outpatient psychiatry clinic with worsening symptoms of depression and suicidal ideation. His history of recurrent MDD spanned over 40 years, first diagnosed in the 1980s. Initially diagnosed with Seasonal Affective Disorder (SAD) during the early stages of his depressive episodes, his diagnosis was later revised to TRD due to a lack of response to oral antidepressants. His depressive episodes were further exacerbated by feelings of being overwhelmed, which were linked to negative work experiences. The patient's depressive symptoms included anhedonia, poor memory, and significant fatigue, leading to reduced involvement in previously enjoyed activities such as gardening, playing tennis, spending time with family, and reading medical journals. During depressive episodes, he became increasingly somatically preoccupied, losing his ability to function and work.

Historically, the patient responded favorably to a single agent for 5-10 years. However, the medication was either tapered or ceased to maintain efficacy over time, requiring dose adjustments or augmentation with an additional agent. In 2012, the patient was on an 11-medication regimen, but did not experience any improvement in depressive symptoms. The patient developed extrapyramidal symptoms in the form of a parkinsonian gait following treatment with risperidone (Risperdal), which necessitated inpatient hospitalization. During this hospitalization, the patient presented with a significant change in mental status characterized by delirium, psychosis, delusions, and a manic-like picture. These symptoms were believed to result, in part, from polypharmacy. Prior to this event, the patient had never exhibited signs of psychosis or frank mania. Under the orders of the attending psychiatrist, atypical antipsychotics, along with all other medications, were discontinued, and the patient underwent 20 sessions of electroconvulsive therapy (ECT). While ECT initially showed a favorable response, recurrent depressive episodes persisted over time. After this hospitalization, the patient was no longer prescribed risperidone (Risperdal) and transitioned to lower-risk antipsychotics. Specifically, olanzapine (Zyprexa) was initiated following ECT in 2012. Later, the patient was switched from olanzapine (Zyprexa) to quetiapine (Seroquel), reflecting a strategy to reduce the potential side effects associated with antipsychotic medications while maintaining psychiatric stability. During this post-ECT period, the patient continued to experience symptoms consistent with MDD, including anxious distress and somatic preoccupation. Notably, there was no evidence of significant cognitive decline immediately following ECT.

In 2017 and 2020, the patient underwent neuropsychiatric evaluations to assess his cognitive abilities, which led to a diagnosis of mild cognitive impairment. The 2017 evaluation revealed several cognitive challenges, including low-average processing speed, poor visual scanning and matching, and difficulty with tasks requiring direction following, which often necessitated repetition. The patient also demonstrated borderline impaired recognition, forgetfulness, and difficulties with cognitive shifting, along with signs of diminished response inhibition. Additionally, there was evidence of increased tremor in the context of a depressed mood. Global functioning was below normal limits, indicating neurocognitive decline. The 2020 evaluation showed subtle improvements in several areas, including processing speed and cognitive flexibility. However, the patient struggled to recall newly learned information and conceptualize it.

Over the years, depressive episodes persisted despite various treatments, with symptoms worsening significantly in August 2022, when the patient experienced his first episode of suicidal ideation, followed by a near attempt during which he ran toward a cliff, requiring inpatient hospitalization for worsening MDD for two months. This event occurred after a shoulder injury in July 2022, which preceded the near suicide attempt and contributed to further restrictions on activities of daily living (ADLs) and daily engagement. Interestingly, lithium had been part of the patient's treatment regimen for the previous four years, but it was discontinued one month prior to this incident. The timing of this discontinuation closely correlates with the onset of the patient's suicidal ideation and near attempt, suggesting a possible link between the abrupt cessation of lithium and the acute worsening of symptoms.

As per family members, the patient had a noticeable decrease in his appetite leading up to the event, with the patient eventually ceasing to eat or drink entirely, only managing to take psychiatric medications with a small amount of water. During this time, the patient faced a significant worsening of MDD with suicidal ideation, which necessitated an additional 20 sessions of ECT. Following this treatment, the patient was described as increasingly confused, delusional, and agitated. Over time, these periods of depression led to greater dependency on his wife for assistance with ADLs and emotional support, highlighting the extensive impact of MDD on his quality of life. 

The patient's medical history includes hypothyroidism, managed with levothyroxine (Synthroid); benign prostatic hyperplasia (BPH), controlled with tamsulosin (Flomax); and hyperlipidemia. The patient has no history of substance abuse or trauma. Over the years, the patient has been prescribed a wide array of oral psychotropic medications, including nortriptyline (Pamelor), sertraline (Zoloft), fluoxetine (Prozac), venlafaxine (Effexor), buspirone (Buspar), aripiprazole (Abilify), quetiapine (Seroquel), risperidone (Risperdal), olanzapine (Zyprexa), lamotrigine (Lamictal), ramelteon (Rozerem), ziprasidone (Geodon), lithium, lorazepam (Ativan), and methylphenidate (Ritalin) as needed. At the time of his presentation to the outpatient psychiatry clinic in December 2023, the patient carried the following diagnoses, which had been established by a neuropsychologist in September 2023 and subsequently confirmed by the geriatric psychiatrist during this consultation: recurrent, treatment-resistant MDD with suicidal ideation (without psychotic features), and mild cognitive impairment. During this initial evaluation, a thorough mental status examination revealed a low mood, diminished interest, and impaired concentration, consistent with severe depression. The patient's Patient Health Questionnaire-9 (PHQ-9) score was 19, indicating severe depressive symptoms. Vital signs were within normal limits, and no significant abnormalities were identified during the initial assessment.

At the initiation of intranasal esketamine therapy, the patient was actively taking the following medications: lithium carbonate (125 mg once daily), quetiapine (25 mg twice daily), desvenlafaxine (150 mg once daily), memantine (10 mg once daily), methylphenidate (20 mg once daily, withheld on treatment days), levothyroxine (100 mcg once daily), and tamsulosin (10 mg once daily). These medications continued throughout the treatment course, with a discussion of medication adjustments below. On January 3rd, 2024, the patient began intranasal esketamine therapy with a four-week induction phase consisting of treatments twice weekly. The first two sessions were dosed at 56 mg, with the following sessions dosed at 84 mg until the induction phase concluded at treatment 8 on January 26th, 2024, with the patient subsequently maintained at 84 mg based on efficacy and tolerability. On January 31st, 2024, the patient began the maintenance phase of intranasal esketamine therapy, with once-weekly sessions until February 12th, 2024. Afterward, the patient continued ongoing maintenance therapy, where the frequency of treatments shifted to once every two weeks until March 25th, 2024, when it transitioned to a monthly schedule for ongoing maintenance. Although some fluctuations marked the treatment timeline, the PHQ-9 score, a widely used tool for assessing depression severity, remained relatively stable despite medication adjustments. At treatment 14, the PHQ-9 score was 4 and remained stable through treatment 15. However, at treatment 16, the PHQ-9 score increased to 5. This shift in score coincided with the emergence of pronounced depressive symptoms, including a growing sense of anhedonia and increased social withdrawal. As a result, before treatment 17, the patient's lithium dosage was adjusted to 150 mg once daily. By treatment 18, the PHQ-9 score had returned to 4, signaling an improvement, with continued progress observed in the following visits. While this brief overview illustrates some of the patient's progress, a more comprehensive breakdown of the PHQ-9, including its scoring criteria and interpretation, is provided in the "Clinical assessments and outcomes" section. This detailed history offers valuable insight into the complexities of managing the patient's struggle with TRD, particularly the challenges of adjusting medication regimens and the impact of long-term ECT treatments. Together, these factors demonstrate the multifaceted nature of his care and the critical importance of continued treatment monitoring to ensure the best outcome.

Treatment plan

**Figure 1:**
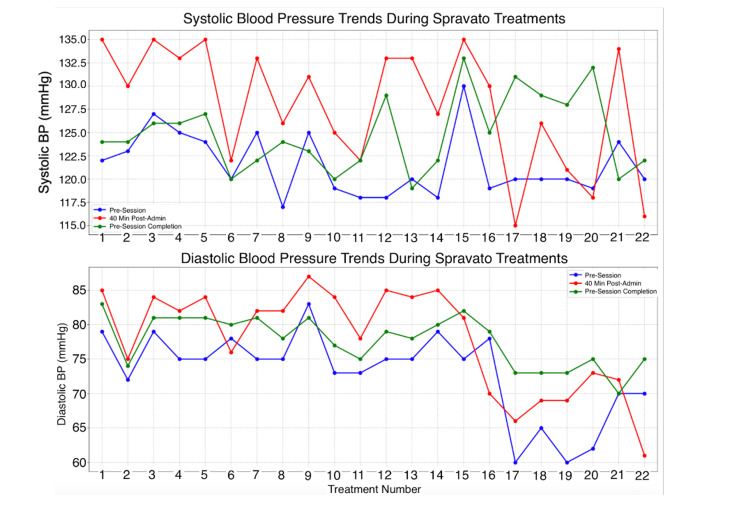
Blood pressure trends during Spravato treatments This figure shows systolic and diastolic blood pressure measured at three-time points: prior to administration, 40 minutes post-administration, and after session completion, illustrating changes in blood pressure throughout the treatment session.

Before initiating treatment, the patient underwent thorough screening to assess eligibility for intranasal esketamine therapy by the requirements of the Spravato REMS (Risk Evaluation and Mitigation Strategy) [9]. This screening included a detailed review of the patient's medical history for contraindications, such as aneurysmal vascular disease, arteriovenous malformation, intracerebral hemorrhage, or hypersensitivity to esketamine or excipients. Additionally, the patient's psychiatric history and current medication use were carefully evaluated to mitigate risks, particularly for individuals with comorbidities like uncontrolled hypertension or cardiovascular disease, as intranasal esketamine has been associated with transient increases in blood pressure and potential cardiovascular risks. Special attention was given to the elderly population due to their higher vulnerability to adverse effects, such as sedation, cognitive impairment, and an increased risk of falls. The administration protocol followed a precise schedule, with one minute between dosing in each nostril and five minutes between each 28 mg applicator. The typically prescribed dosages for intranasal esketamine are as follows: each nasal spray applicator contains 28 mg of ketamine, and for a 56 mg dose, two sprays of 14 mg are administered in each nostril. For an 84 mg dose, three sprays of 14 mg are administered in each nostril. Following each dose, patients, especially those in higher-risk age groups, remained under supervision for at least two hours to monitor for potential adverse effects, including increased blood pressure, dissociation, and sedation. The patient's vital signs, including blood pressure and heart rate, were recorded before the treatment session, 40 minutes post-administration, and at session completion to ensure hemodynamic stability (Figure 1). The patient experienced mild to moderate adverse effects during treatment, all of which improved with subsequent sessions after supportive care modifications. Intranasal esketamine therapy typically involves multiple sessions, with dosing frequency and number of sessions tailored based on patient response, particularly for elderly patients who may require adjustments due to increased sensitivity to side effects.

**Figure 2:**
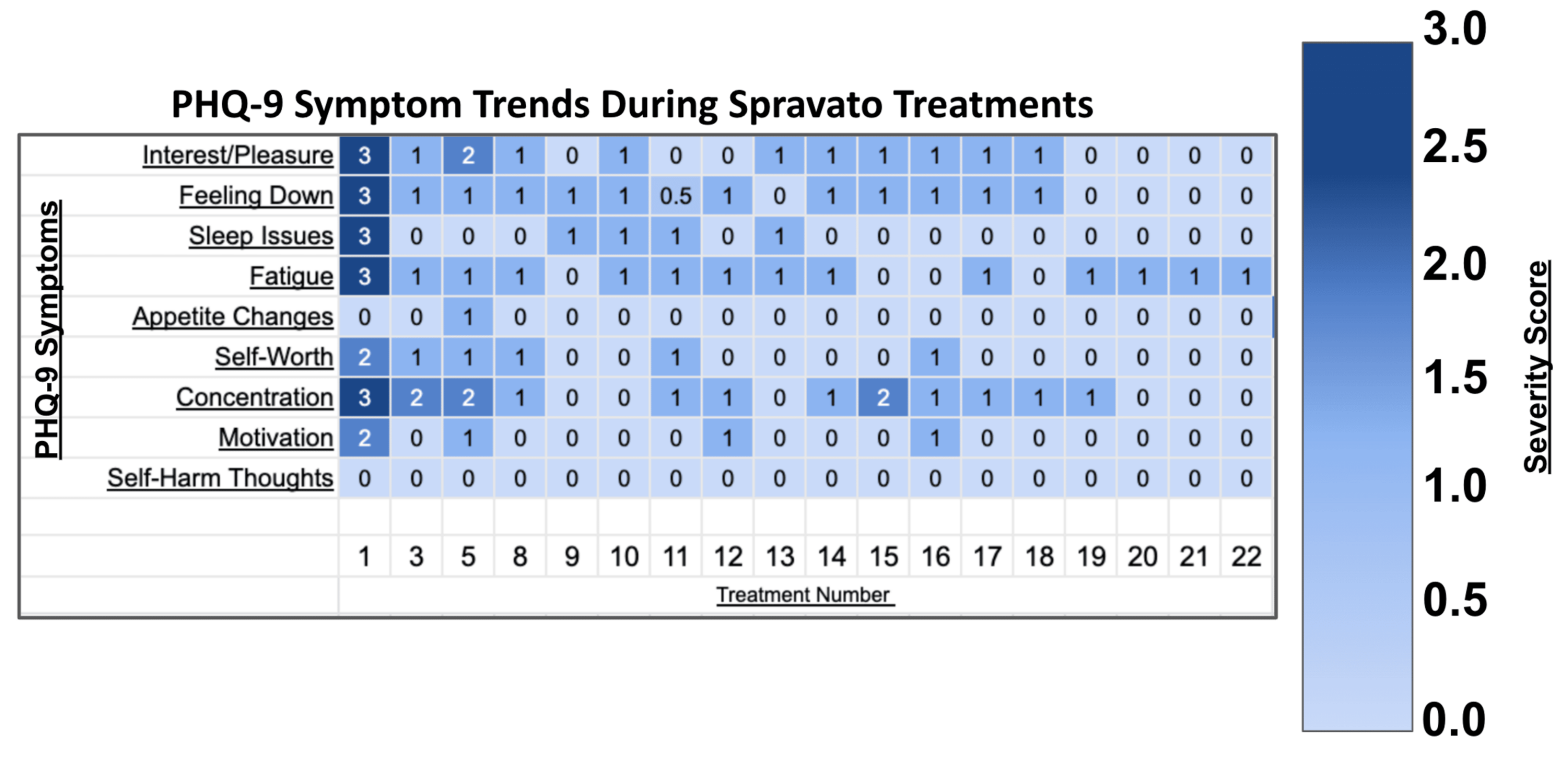
PHQ-9 symptom trends during Spravato treatments This figure illustrates the progression of PHQ-9 symptom severity across treatment sessions. The rows represent each PHQ-9 symptom: interest/pleasure, feeling down, sleep issues, fatigue, appetite changes, self-worth, concentration, movement/speech, and self-harm thoughts, while the columns track individual treatment sessions. Darker colors indicate higher symptom severity, with lighter colors reflecting improvement over time. PHQ-9, Patient Health Questionnaire-9

Lightheadedness was this patient's first significant adverse effect at treatment 5. The patient had a brief, two-minute episode of moderate lightheadedness following the treatment, which required a 15-minute extension of the two-hour post-treatment observation period for the adverse effect to diminish. As a precaution, this extended observation was maintained in subsequent treatments; however, the adverse effect was not observed after the initial episode and subsided without further intervention. Mild disorientation was reported, particularly during treatments 2 and 3 during the induction phase, experienced shortly after administration while seated in the treatment chair. After adjusting his position, the symptoms started to resolve, and once the chair was reclined back, disorientation diminished and did not recur in subsequent sessions. Mild sedation was another reported adverse effect, typically occurring post-treatment. The patient described a relaxed state but remained alert, oriented to time, person, and place, and responsive to verbal commands. Initially, sedation persisted for the remainder of the day following treatment, but this effect gradually subsided by the next morning. As treatments progressed, the sedative effects diminished. By the start of the maintenance phase, at treatment 9 on January 31st, 2024, the patient reported feeling relaxed but fully awake, with no impact on daily activities. The patient first reported moderate double vision during treatments 3 and 4, occurring for a 5-10 minute period following intranasal esketamine administration. In response, the care team provided an eye mask at treatment 5, which the patient found effective in reducing the frequency and severity of the symptom. As treatment progressed, the frequency and impact of double vision decreased, and by treatment 16, the patient no longer reported experiencing the symptoms. While the eye mask remained available, the patient indicated it was no longer necessary. Lastly, mild nasal irritation was another adverse effect, occurring briefly after intranasal administration and subsiding naturally within minutes after each administration. No further intervention was necessary for the nasal irritation. By treatment 13, the adverse effects had either subsided or become significantly less impactful, allowing the patient to proceed comfortably with maintenance therapy. No severe adverse events occurred during intranasal esketamine treatment. By the end of each treatment session, after any adverse reactions had subsided, the patient felt well enough to attend the senior center with his Home Health Aide (HHA) and continue participating in scheduled weekly social activities. While mild adverse effects occurred, the patient's overall functioning and ability to engage in daily routines remained largely unaffected, indicating a positive response to therapy. By the 13th treatment session, significant improvements in depressive symptoms, as reflected by PHQ-9 scores (Figure 2), supported the decision to transition the patient to ongoing maintenance intranasal esketamine therapy.

Clinical assessments and outcomes

The patient presented with recurrent, severe MDD, with a history of suicidal ideation and suicide attempts, as well as significant impairment in daily functioning and cognitive decline. After extensive neurological and psychological testing in 2017 and 2020, including the Beery Visual-Motor Integration (VMI) Developmental Test of Visual Perception, the Behavior Rating Inventory of Executive Function (adult version), the California Verbal Learning Test (second edition), and the Wechsler Adult Intelligence Scale (fourth edition), among others, it was determined that cognitive decline was attributed to the severity of the patient's depression, rather than any underlying neurological condition. Testing revealed that his overall intellectual functioning was average, with above-average verbal abilities. His perceptual reasoning was within normal limits but showed variability between tasks. Global functioning was categorized as "below average but intact," with clear indications of neurocognitive dysfunction, particularly in memory and executive functioning, which seemed to reflect a decline from prior performance levels. Despite some memory difficulties, particularly in recalling newly learned information, his recognition abilities were better preserved, suggesting challenges with retrieval rather than encoding. Additionally, while abstract reasoning and set-shifting abilities were intact, difficulties with cognitive flexibility, as evidenced by perseveration during a conceptual reasoning task and diminished response inhibition, were demonstrated. These findings were consistent with a diagnosis of mild neurocognitive disorder, likely exacerbated by the severity of his depression.

Before each intranasal esketamine treatment, the patient completed a PHQ-9, a self-reported assessment tool used to evaluate the severity of depressive symptoms. These questions aim to assess the patient's frequency of the most common depression symptoms over the last two weeks. The PHQ-9 assesses symptoms commonly associated with depression, including diminished interest in activities, feelings of hopelessness or sadness, sleep disturbances, low energy, changes in appetite or eating habits, feelings of worthlessness, difficulty concentrating, psychomotor slowing, and thoughts of self-harm or suicide. PHQ-9 scores of 5, 10, 15, and 20 represent mild, moderate, moderately severe, and severe depression, respectively [11]. On the initial day of intranasal esketamine treatment, the patient's self-reported PHQ-9 score was 19, showing signs of a severely depressed mood. The patient reported having little interest in doing things, low energy, feelings of hopelessness, lack of appetite, trouble concentrating, and suicidal ideation nearly every day. Cognitive impairment further hindered his daily functioning, making it difficult to complete daily tasks and responsibilities without the help of his wife, often spending much of this time in bed. By treatment 5, the patient's self-reported PHQ-9 score had decreased to 9, with the patient reporting a lack of interest in doing things, depression, low energy, a poor appetite, and difficulty concentrating on only some days now, as opposed to nearly every day before intranasal esketamine therapy. The patient also stated that he no longer had suicidal ideation. By treatment 8, the patient reported a significant improvement in mood, describing feelings of hope for the future. At this point, he was engaging in basic daily activities, such as walking, physical therapy, and spending time at the senior center several times weekly. His cognitive abilities also began to improve. Specifically, he demonstrated enhanced attention and processing speed, allowing him to complete previously overwhelming tasks. By the 14th treatment, his PHQ-9 score had decreased to 4. At this point, the patient's only reported symptoms were a lack of interest in doing things, feelings of depression, low energy, and difficulty concentrating, which he reported experiencing only on certain days. In his most recent 22nd treatment in November 2024, the patient had a self-reported PHQ-9 score of 1, with the only remaining symptom being low energy on some days. This marked a significant improvement in symptom severity and frequency since beginning intranasal esketamine treatment. In addition to his mood improvement, the patient's cognitive function showed significant progress. He reported better memory and recall, enabling him to engage more fully with his environment and interactions. His executive function improved, demonstrated by his ability to plan and organize tasks independently. The patient continued participating in daily activities, such as gardening and attending the senior center four times weekly, where he was self-motivated. This sustained improvement in mood, cognition, and activity further demonstrates the reduction in his depression symptoms following intranasal esketamine therapy. The patient is currently continuing intranasal esketamine therapy every month with no changes to his treatment regimen. He remains on the same medications as listed previously, and his condition has remained stable, with ongoing improvements in both mood and cognitive function. The patient's continued engagement in daily activities and his stable mental health reflect the positive outcomes of his treatment plan.

## Discussion

Treating the geriatric population with TRD can be clinically challenging due to various factors. Adverse effects associated with intranasal esketamine therapy typically include dissociation, dizziness, nausea, and sedation. Although less common, serious side effects may occur, such as psychotic symptoms, urinary tract issues, and respiratory depression. Elderly patients are particularly at risk for cognitive disturbances and falls due to sedation and dissociation, necessitating careful monitoring during treatment. The geriatric population is usually more medically complex than their younger TRD counterparts. Older TRD patients typically have other medical comorbidities aside from their depression, hindering them from receiving treatment sooner [3,4]. These conditions include, but are not limited to, a history of cerebrovascular or cardiovascular incidents, age-related cognitive impairment, essential hypertension, liver disease, and aneurysms. Liver disease is a lesser-known relative contraindication, as impaired hepatic function can affect drug metabolism, leading to slower clearance of esketamine from the body and potentially increased plasma concentrations [9].

Another example, relevant to this case, is tamsulosin (Flomax), a medication for BPH. Approximately 50% of men over the age of 65 have some form of BPH and are prescribed tamsulosin, an alpha-1A antagonist. This medication blocks smooth muscle receptors of the prostate and urethra to ameliorate urine flow. However, this alpha-1A antagonism of tamsulosin also increases these patients' risk for falls [12]. Patients concurrently taking tamsulosin and undergoing intranasal esketamine therapy face an elevated risk of falls due to potential effects on blood pressure and balance. As a result, our treatment protocol was adapted to include an extended monitoring period for this patient, which was subsequently implemented for all geriatric patients receiving intranasal esketamine therapy.

Limited published clinical trials have been done on this population and should be explored further, including drug-drug interactions. Geriatric populations are prescribed opioids at a much higher rate than younger individuals due to the increased need for adequate pain control of other ailments that younger people typically do not suffer from. Combined use of opioids and intranasal esketamine can put these patients at increased risk of respiratory depression, sedation, and falling risk [7,13]. A phase 1 open-label, single-dose pharmacokinetic study comparing healthy elderly patients (≥65 years of age) to healthy younger adults (≥18 to ≤55 years of age) showed that the plasma Cmax and area under the curve (AUC) of esketamine after a single 28-mg intranasal dose were 17% to 21% higher in geriatrics compared to younger adult patients [14]. These findings indicate that intranasal esketamine exhibits augmented pharmacokinetic ability in geriatric populations, resulting in typically higher plasma drug concentrations than younger adults. Consequently, geriatric patients may experience more pronounced or prolonged effects, underscoring the need for tailored monitoring protocols and dosage adjustments to minimize potential adverse reactions.

**Figure 3:**
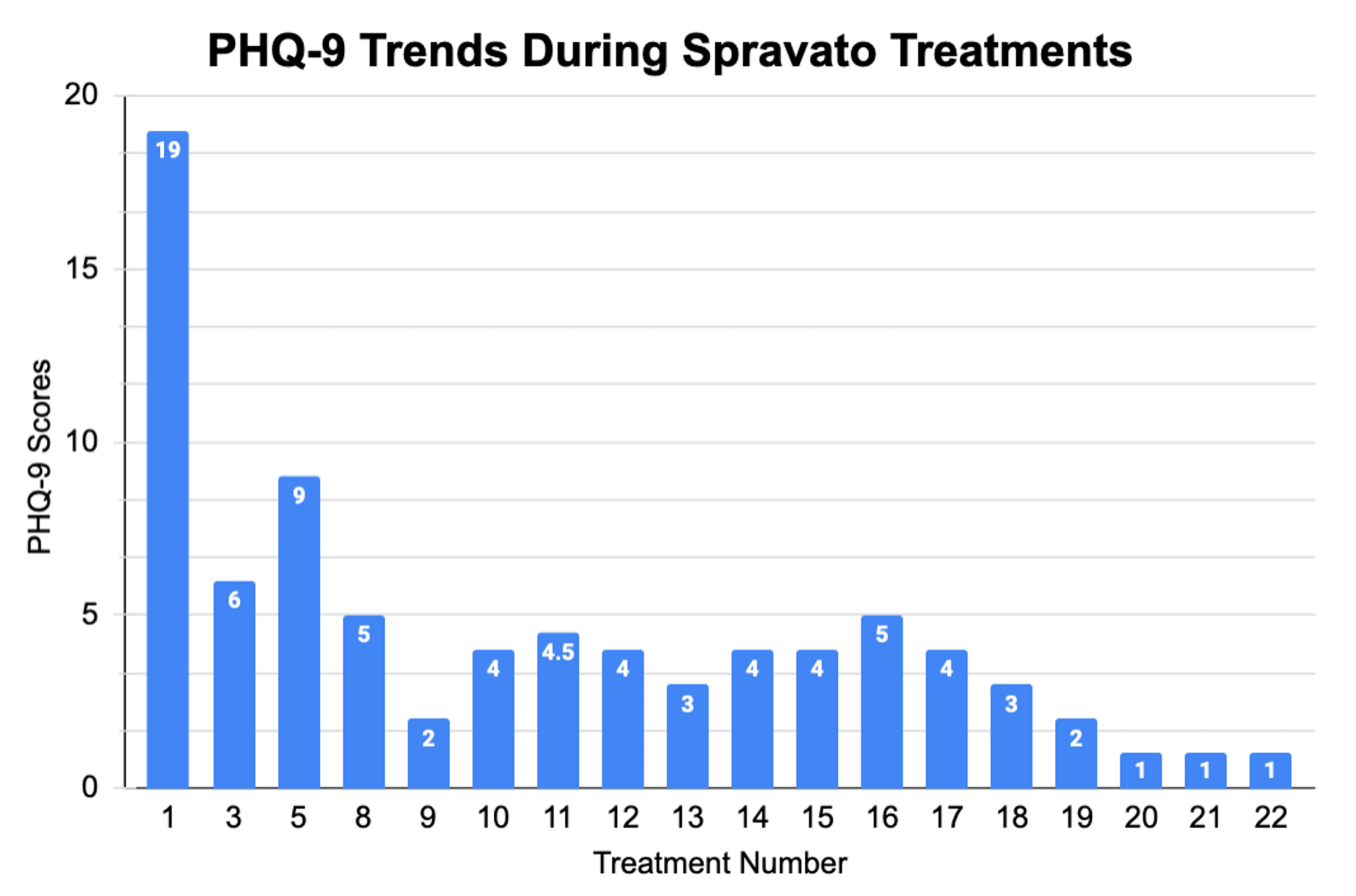
PHQ-9 scores during Spravato treatments This figure shows overall PHQ-9 scores across 18 assessments during Spravato treatment, highlighting a sharp decline after the initial session and a continued reduction in depressive symptoms over time. PHQ-9, Patient Health Questionnaire-9

In the SUSTAIN-2 phase 3 trial, the efficacy and safety of intranasal esketamine were compared between younger (18-64 years) and older (≥65 years) patients with TRD. They exhibited a similar reduction in Montgomery-Åsberg Depression Rating Scale (MADRS) scores in both the induction and optimization/maintenance phases, with similar numbers of reported serious treatment adverse events [15]. While the phase 3 short-term, flexible-dose clinical trial (TRANSFORM-3) demonstrated clinically significant, though not statistically significant, improvements in MADRS scores from baseline to day 28 compared to the oral antidepressant + placebo nasal spray (AD + PBO) group, further research is warranted to evaluate intranasal esketamine's efficacy and safety in elderly populations [8]. In contrast, this case report highlights a remarkable reduction in depressive symptoms, as evidenced by a PHQ-9 score improvement from 19 at baseline to 1 by the 22nd treatment, reflecting a symptom reduction of over 75% (Figure 3).

## Conclusions

A limitation of this case study is its single-patient design, which restricts the generalizability of the findings to the broader geriatric population. While the positive response observed in this individual case is promising, further research with larger sample sizes is necessary. Clinicians treating treatment-resistant MDD in geriatric populations should consider intranasal esketamine therapy as a viable option, especially for those unresponsive to conventional therapies. To ensure safety and maximize benefits, clinicians should implement extensive safety and monitoring protocols during and after treatment sessions to address potential adverse effects, such as transient disorientation. Additionally, clinicians should consider individualized dose adjustments, tailored to the unique pharmacodynamic challenges of geriatric patients, particularly those with comorbidities or on multiple medications.

In conclusion, Spravato (intranasal esketamine) led to a significant reduction in the frequency and severity of symptoms associated with treatment-resistant MDD, and an increase in quality of life, with minimal and manageable adverse effects. Exemplified by an 86-year-old with a complex medical and psychiatric history, this study presents valuable insights into the safety and efficacy of intranasal esketamine therapy as an effective option for TRD in geriatric populations. Therefore, the study recommends that further research be performed for this demographic, as the findings align with the data presented, further supporting the potential of intranasal esketamine as a therapeutic option for TRD in geriatric populations. Future research should prioritize long-term studies to validate sustained efficacy and safety, refine dosing guidelines, and identify clinical markers that can predict patient responses. These efforts are crucial in integrating intranasal esketamine therapy into standardized treatment guidelines, ultimately enhancing the prognosis for geriatric patients with TRD.

## References

[1] American Psychiatric Association (2013). Diagnostic and Statistical Manual of Mental Disorders (5th ed). American Psychiatric Association.

[2] Jalali A, Ziapour A, Karimi Z (2024). Global prevalence of depression, anxiety, and stress in the elderly population: a systematic review and meta-analysis. BMC Geriatr.

[3] Jaros A, Rybakowski F, Cielecka-Piontek J (2024). Challenges and opportunities in managing geriatric depression: the role of personalized medicine and age-appropriate therapeutic approaches. Pharmaceutics.

[4] Raue PJ, McGovern AR, Kiosses DN, Sirey JA (2017). Advances in psychotherapy for depressed older adults. Curr Psychiatry Rep.

[5] Barney LJ, Griffiths KM, Christensen H, Jorm AF (2009). Exploring the nature of stigmatising beliefs about depression and help-seeking: implications for reducing stigma. BMC Public Health.

[6] Lotrich FE, Pollock BG (2005). Aging and clinical pharmacology: implications for antidepressants. J Clin Pharmacol.

[7] (2024). Spravato (esketamine) medication guide. https://www.janssenlabels.com/package-insert/product-monograph/prescribing-information/SPRAVATO-pi.pdf.

[8] Ochs-Ross R, Daly EJ, Zhang Y (2020). Efficacy and safety of esketamine nasal spray plus an oral antidepressant in elderly patients with treatment-resistant depression - TRANSFORM-3. Am J Geriatr Psychiatry.

[9] (2024). Center for drug evaluation and research application. https://www.accessdata.fda.gov/drugsatfda_docs/nda/2019/211243Orig1s000REMS.pdf.

[10] Daly EJ, Trivedi MH, Janik A (2019). Efficacy of esketamine nasal spray plus oral antidepressant treatment for relapse prevention in patients with treatment-resistant depression: a randomized clinical trial. JAMA Psychiatry.

[11] Ford J, Thomas F, Byng R, McCabe R (2020). Use of the Patient Health Questionnaire (PHQ-9) in practice: interactions between patients and physicians. Qual Health Res.

[12] Welk B, McArthur E, Fraser LA, Hayward J, Dixon S, Hwang YJ, Ordon M (2015). The risk of fall and fracture with the initiation of a prostate-selective α antagonist: a population based cohort study. BMJ.

[13] Mikelyte R, Abrahamson V, Hill E, Wilson PM (2020). Factors influencing trends in opioid prescribing for older people: a scoping review. Prim Health Care Res Dev.

[14] (2024). An open-label, long-term, safety and efficacy study of intranasal esketamine in treatment-resistant depression. Esketamine. Clinical Study Report ESKETINTRD1012. Janssen Research & Development, LLC. EDMS-ERI-118138889.

[15] Ochs-Ross R, Wajs E, Daly EJ (2022). Comparison of long-term efficacy and safety of esketamine nasal spray plus oral antidepressant in younger versus older patients with treatment-resistant depression: post-hoc analysis of SUSTAIN-2, a long-term open-label phase 3 safety and efficacy study. Am J Geriatr Psychiatry.

